# On the possible cause of distinct El Niño types in the recent decades

**DOI:** 10.1038/srep17009

**Published:** 2015-11-24

**Authors:** Jyoti Jadhav, Swapna Panickal, Shamal Marathe, K. Ashok

**Affiliations:** 1 Centre for Climate Change Research, Indian Institute of Tropical Meteorology, India; 2 University of Hyderabad, Hyderabad, India; 3 Indian Institute of Tropical Meteorology, Pune, 411008, India

## Abstract

Distinct El Niño types have been observed in the recent decades with warm anomalies in the eastern Pacific (Canonical El Niño, EL) and central Pacific (El Niño Modoki, EM). Among these, a basinwide tropical Pacific (TP) warming is seen during 2009 and recently during 2014. We carried out data analysis and numerical simulation experiments to understand the possible cause for different El Niño flavours. The results reveal that the co-evolution of ocean-atmospheric conditions are critically important. Stronger boreal spring (Mar-May) through summer (June-September) westerly wind anomalies (WWA), with relatively stronger ocean pre-conditioning can lead to EL, weaker ocean pre-conditioning and weaker WWA can generate EM, while stronger ocean preconditioning and weaker WWA can lead to basinwide warming pattern. The strength of the WWA is crucial in determining the strength of the ocean dynamic response and the thermocline displacements in the Pacific. The study has important implications for understanding the nature of El Niño in advance.

The El Niño–Southern Oscillation (ENSO) is the dominant mode of interannual variability in the tropical Pacific having global socioeconomic impacts^1^,^2^,^3^,^4^. The canonical El Niño^4^,^5^,^6^ (canonical El Niño, hereafter EL), is typified by warm sea surface temperature anomaly (SSTA) in the eastern tropical Pacific and cold anomalies in the west. In recent decades we see an increasing frequency of the El Niño Modoki (EM) events with warm SSTA in the central equatorial Pacific flanked by colder SSTA on both sides^7^. Notwithstanding the different nomenclatures employed for the EM (e.g., central pacific/warm tongue events) and EL (e.g., eastern pacific/cold tongue) events, most of the studies^7^,^8^,^9^,^10^,^11^,^12^,^13^,^14^ have commonly identified that EM events, and their evolution and impacts, are different from EL. Interestingly, during June-September of 2009, a hitherto unforeseen basinwide warming pattern in the tropical Pacific (hereafter TP) was observed^15^.

On a different note, the similarities between the oceanic conditions in the tropical pacific in early 2014 were similar to that in 1997, suggesting that an El Niño matching the 1997 intensity may develop during 2014; however we see basinwide warming pattern during boreal spring (Mar-May) and again during September through December 2014. Since the pattern of the tropical pacific SSTA has wide impact on the global weather and climate and different El Niño types have different global teleconnections, understanding the nature and evolution of different El Niño types is vital.

A flattening of the thermocline in the equatorial Pacific caused by a weakening of equatorial easterlies in response to global warming was suggested to be one of the reasons for the observed changes in the characteristics of El Niño events in the recent decades^7^,^14^. However, the character of El Niño events itself has varied naturally in the recent decades^16^. The positive feedback between ocean and atmosphere through Bjerknes feedback is essential for the growth of an El Niño. The intensity of Bjerknes feedback and the recharge-discharge processes are found to be different for different El Niño types^17^,^18^,^19^,^20^,^21^,^22^. These motivate an important question, specifically, what drives the distinction between different El Niño types.

The changes in the warm water volume (WWV) in the equatorial Pacific was considered to be a good indicator for the development of an El Niño^23^, but recent events have shown that the preconditioning of the ocean with development of WWV itself does not generate an El Niño^16^,^24^. A recent study^25^ also indicates that subsurface processes of discharging warm water is preconditioned eighteen months before the mature phase of an El Niño, though no specific distinction can be noticed in the subsurface ocean conditions between different El Niño types at this juncture. The potential importance of the high frequency wind variability associated with westerly wind events (WWE) on triggering the El Niño events has been emphasized by many studies^24^,^26^,^27^,^28^,^29^. The intensity of WWE also modulates the strength and timing of El Niño events^27^,^30^,^31^. Studies^24^, have reported that the lack of WWE during boreal spring was considered to be the prime reason for the distinction of 2014 event from 1997 El Niño. However, a WWE alone cannot generate an El Niño. Therefore, in this paper, we carry out a detailed analysis of the observed and reanalyzed datasets to explore if the generating mechanisms for different El Niño types are different from one another. To substantiate our findings from the analysis, we also carried out several numerical simulation experiments with an ocean model to assess the relevance of the oceanic pre-conditioning for El Niño, that of the strength of the atmospheric circulation, and their combinations.

## Results

### Distinct evolution of El Niño flavours

**Figure 1:**
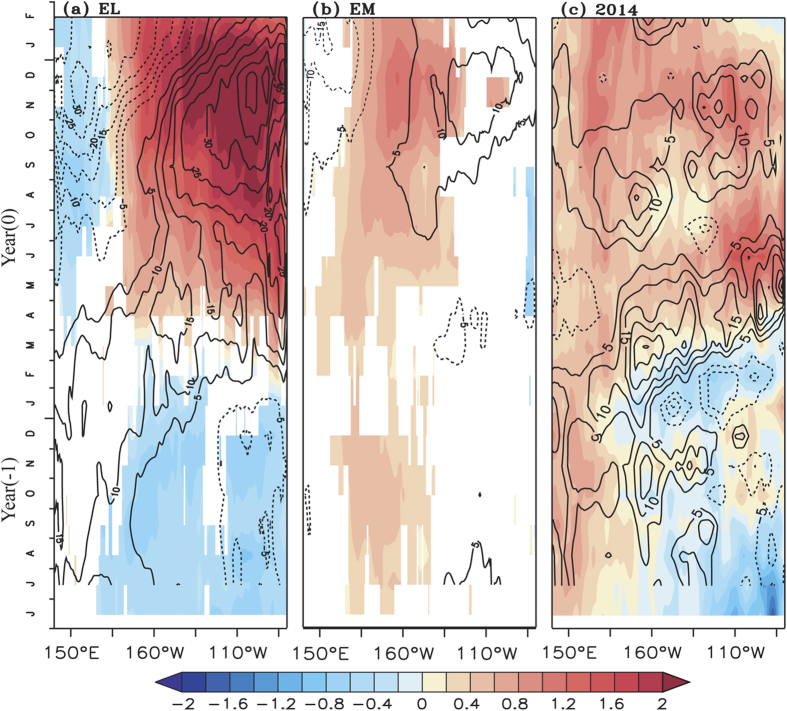
Time-longitude plot showing composite anomalies of sea surface temperature (°C, shaded) and 20 °C isotherm depth (m, contour) averaged between 5°S-5°N for (**a**) canonical El Niño (**b**) El Niño Modoki. Significance values above 90% confidence level from a two-tailed student’s t-test are shaded in (**a**) and (**b**) and (**c**) 2014 anomalies of sea surface temperature (°C, shaded) and 20 °C isotherm depth (m, contour) averaged between 5°S-5°N. Being single case, the values without statistical significance is shown for 2014.

Composites of equatorial SSTA and thermocline depth (D20) anomalies (Fig. 1) during EL, EM and TP years show appreciable differences in the spatial pattern and seasonality during the evolution. The anomalies above 90% confidence level from a two-tailed student’s t-test are shown for EL and EM in Fig. 1a,b. The amplitude of SST anomalies during the EM events is weaker as compared to EL. The warm SSTA are confined to the central equatorial Pacific, flanked by colder SSTA on both sides, in late boreal spring (Mar-May) of the EM events (refer as year (0)) (Fig. 1b). These warm SSTA in the central tropical pacific grow and peak by late boreal fall (Oct-Nov) of year (0). We also see a weak, and largely statistically insignificant, expansion of the warming towards east. The anomalies in the east weaken by the latter half of the boreal winter (Jan-Feb), unlike in the central Pacific where the warming sustains through another season. On the other hand, for the EL events (Fig. 1a), the warm SSTA are seen in the central and eastern equatorial Pacific by late boreal spring and amplify during the consequent seasons, through boreal winter.

On the other hand, the SST anomalies for 2014 and 2009 shaded in Fig. 1c and Fig. S1a respectively, shows the warm SSTA in the western and central equatorial Pacific by boreal spring. We see an eastward extension of warm anomalies since late boreal spring, without cooling the western Pacific unlike an EL or an EM event. Thus a basinwide warming pattern persists in the tropical Pacific from late boreal spring till the decay of the event. To test the significance of the SSTA during TP events, we have presented SST departure (difference of SST between TP and each of EL, EM and EL & EM) in Fig. S2. The SST departure above 90% confidence level from a two-tailed student’s t-test is shaded in Fig. S2. The 2014 and 2009 TP events are considered in Fig. S2. It can be seen from the figure that the spatial pattern of SST departure is entirely different from SSTA for EL and EM indicating that TP event is different from EL and EM.

We note that many studies^32^,^33^ have categorised the tropical Pacific conditions during 2009 winter as a strong El Niño Modoki case. Indeed, the SSTA in the tropical Pacific looks like a strong EM signal during that season. However, the magnitude of the EMI during the summer of the 2009 falls below the threshold^11^,^34^, and thus does not meet the important criterion of persistence of Modokis, which separates the Modokis from the trans Niño signals^35^ associated with the canonical El Niño evolution. Therefore we would not categorise the 2009 event as a complete cycle of the El Niño Modoki event. Considering the two TP events, defining an index for TP is not appropriate, however, we can tentatively suggest a TP event to have occurred if positive SST anomalies persist from west-east in the western and eastern tropical Pacific boxes (90°W-80°W, 5°S-5°N) and (125°E-145°E, 5°S-5°N) for two consecutive seasons of boreal summer (JJA) and boreal fall (SON). Importantly, TP (green) EL (red) and EM (blue) can be seen from the scatter plot of SST anomalies averaged in eastern and western Pacific boxes as shown in Fig. S6. A clear separation of the EL (red) and EM (blue) can be seen, suggesting the distinctness of the two flavours in agreement with earlier works^13^,^34^. During TP events the SST anomalies are positive in these boxes for two consecutive seasons of JJA and SON. However, during EL and EM, SST anomalies are either negative or positive, does not persist for these two consecutive seasons.

The distinct SST pattern during different El Niño types is corroborated by distinct thermocline response as shown by the D20 anomalies (contour in Fig. 1). The thermocline in the eastern tropical Pacific starts deepening by boreal summer along with a shoaling in the western equatorial Pacific during an EL (Fig. 1a), and is anomalously deeper by about 30m in the east during the mature phase (boreal winter (Dec-Feb)). A sharp east-west gradient is generated in the equatorial thermocline anomaly, which amplifies the Bjerknes feedback^36^ and warms the SST in the eastern Pacific (Fig. 1a). On the other hand, during an EM, the eastern thermocline is comparatively shallow, with a maximum deepening of only about 10m is seen in the east and a weak shoaling in the west (Fig. 1b) during its peak phase. The anomalous peaks in the SSTA and D20 during EM events are not co-located as in case of an EL event. Thus the east-west thermocline gradient is weaker during EM as compared to EL and weaker SSTA is seen in the central and eastern Pacific during the mature phase. However during a TP event (Fig. 1c & S1a), the thermocline deepened in the east during boreal summer, and was comparable to EL (Fig. 1a). Similarly the shoaling in the west is also weaker. Thus, a very weak east-west gradient is generated across the equatorial Pacific even during the mature phase of a TP event.

### Impact of westerly wind anomalies on El Niño evolution

**Figure 2:**
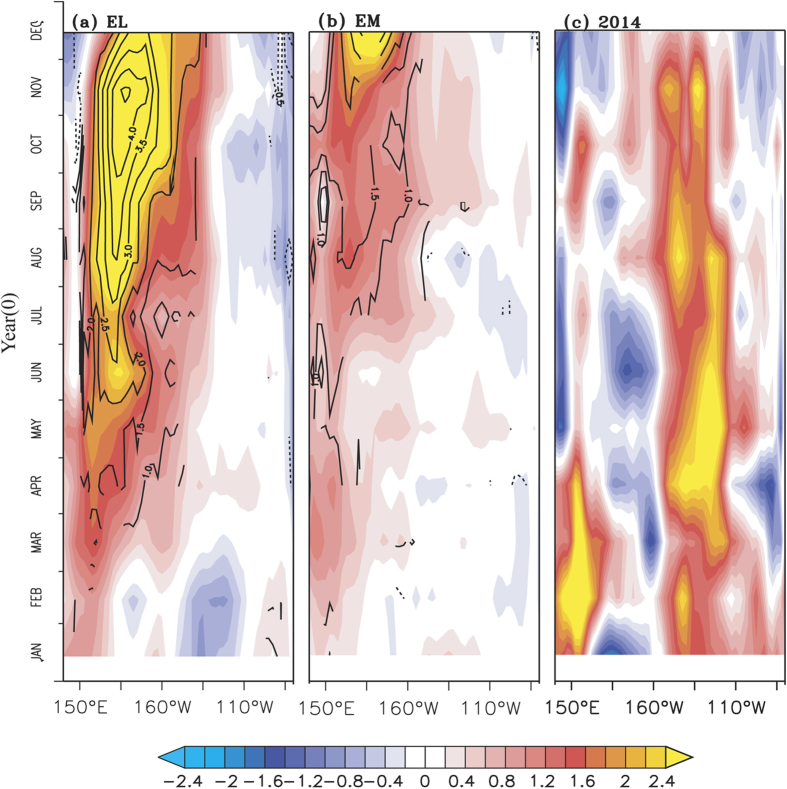
Time-longitude plots of zonal wind anomalies (m s^−1^) averaged between 5°S-5°N for (**a**) canonical El Niño (**b**) El Niño Modoki, significance values above 90% confidence level from a two-tailed student’s t-test are shown as contours in (**a–c**) zonal wind anomalies (m s^−1^) averaged between 5°S-5°N for 2014. Being single case, the values without statistical significance is shown for 2014.

The difference in the evolution of thermocline and SSTA can be explained by analysing the zonal wind anomalies over the equatorial Pacific during El Niño years. Numerous studies have shown the importance of westerly wind anomalies (WWA) on the dynamical response of the ocean to generate the downwelling Kelvin waves^22^,^37^,^38^,^39^,^40^,^41^,^42^, and more recently in the context of the El Niño types^43^. The zonal wind anomalies averaged over the equatorial Pacific between 5°S-5°N for different tropical pacific ‘warming event’ are shown in Fig. 2; the anomalies above 90% confidence level from a two-tailed student’s t-test are contoured for the EL and EM events. Strong WWA can be noticed from boreal spring of an EL over the western to central equatorial Pacific, with intensity increasing from boreal spring to boreal fall (Fig. 2a). We confirm the relatively strong westerly anomalies during the EL events by leaving out the extreme EL events of 1982 and 1997 in composite analysis of the zonal wind anomalies (Fig. not shown). While during EM, the WWA are significantly weaker as compared to EL events, and confined to the western part of the basin (Fig. 2b). During TP events, WWA were absent, during boreal summer of 2014 (Fig. 2c) and boreal spring of 2009 (Fig. S1b) and weak WWA in the western to central Pacific are seen during rest of the period (Fig. 2c & S1b); interestingly, we see anomalous easterlies in the western Pacific during boreal summer of 2014 and boreal spring of 2009. The anomalous westerlies over the western and central equatorial Pacific are known to generate downwelling Kelvin waves^44^,^45^,^46^, which propagate along the thermocline into the eastern Pacific. The downwelling Kelvin wave propagation is evident from the deepening of thermocline as shown by the positive D20 anomalies (Fig. 1). As compared to weaker WWA and D20 anomalies during EMs (Figs 1b & 2b), stronger WWA (Fig. 2a) during EL events generate stronger thermocline response (Fig. 1a) as evident from the deeper D20 anomalies.

**Figure 3:**
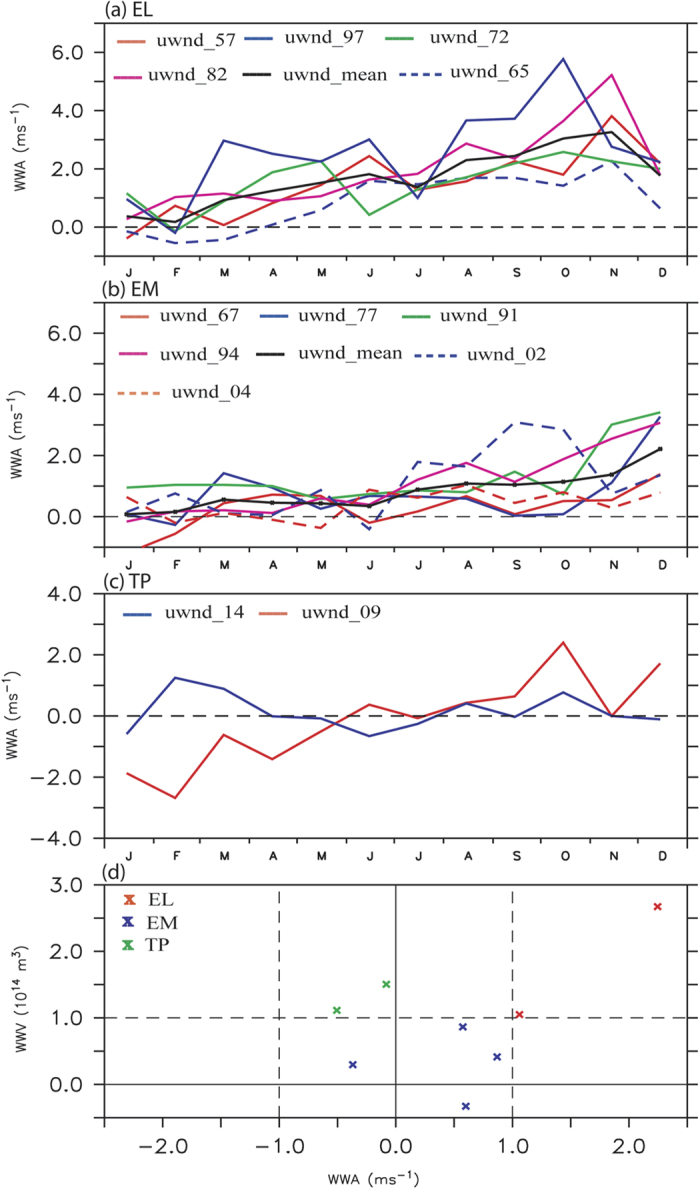
Time series showing the seasonal evolution of zonal wind anomalies (m s^−1^) over equatorial western Pacific (150°E-160°W, 5°S-5°N) region for (**a**) canonical El Niño (**b**) El Niño Modoki and (**c**) 2009 and 2014. (**d**) Scatter plot of western equatorial Pacific zonal wind anomalies (ms^−1^, 150°E-160°W, 5°S-5°N) and WWV anomalies (10^14^ m^3^) during late boreal spring (May) in tropical Pacific for EL (Red), EM (Blue) and TP (Green). The values above one standard deviation are shown as dashed line.

Another crucial aspect is the seasonality of WWA in the western-central Pacific. Triggering of eastward currents and generating equatorial downwelling oceanic Kelvin waves that propagate to the east and deepen the thermocline in the central and eastern equatorial Pacific takes place only in a particular multi-month window^29^. Indeed, the seasonality in the evolution of anomalous westerlies is also different between different El Niño types (Fig. 3a,b). Zonal wind anomalies are averaged in the western equatorial Pacific (150°E-160°W, 5°S-5°N) and are shown in Fig. 3a–c. The anomalous westerlies with a mean strength of 1 ~ 1.5 m s^−1^ (as shown by thick black line) are seen in boreal spring during an EL (Fig. 3a), and they amplify during boreal summer through fall to a maximum intensity of about 4 m s^−1^. However, the anomalous westerlies during an EM are weaker, with a mean intensity of about 0.5 m s^−1^ seen during boreal spring and 1 m s^−1^ during boreal summer through fall (Fig. 3b). During the two TP events, anomalous equatorial westerlies are absent during boreal summer of 2014 and during boreal spring of 2009 (Fig. 3c), supported by Figs. 2c & S1b. Studies^47^ noted the absence of boreal summer westerlies during 2014.

The importance of the late boreal spring (May) wind intensity, and that of the ocean pre-condition for the El Niño evolution is shown in Fig. 3d. The magnitude of zonal mean warm water volume (WWV, 10^14^ m^3^) anomalies in the equatorial Pacific (120°E-80°W, 5°N-5°S) is an important parameter related to El Niño evolution, as discussed by Meinen & McPhaden^23^, Who found that amplitudes of WWV anomalies are linearly related to the amplitudes of the Niño-3 (150°W-90°W, 5°S-5°N) SSTA, with larger anomalies in WWV preceding larger anomalies in SST by seven months. This indicates that ocean gets preconditioned by late boreal spring (May) before the mature phase of a canonical El Niño. To explore further, we plot the anomalies of zonal wind averaged between 150°E-160°W; 5°S-5°N and WWV anomalies for late boreal spring (May, two seasons prior to mature phase of El Niño) in Fig. 3d. The values above one standard deviation are shown as dashed line in the figure. The WWV anomalies from TAO data is available since 1980, the events after 1980 are shown in the figure. Larger WWV anomalies (Fig. 3d) are observed prior to an EL and weaker anomalies prior to EM indicating the strength of pre-conditioning of equatorial Pacific during these events. During the TP events, the pre-conditioning of the ocean is similar to EL. The apparent distinction of EM from EL and TP is in the ocean pre-condition, as shown by WWV anomalies below 1 standard deviation for EM events. *Importantly, though the ocean pre-condition is the same for EL and TP the zonal wind anomalies are westerly for EL and easterly for TP thus making EL and TP evolution different.* The ocean sub-surface evolution during different El Niño flavours is explored in the following section.

### Ocean dynamic response during different El Niño flavours

**Figure 4:**
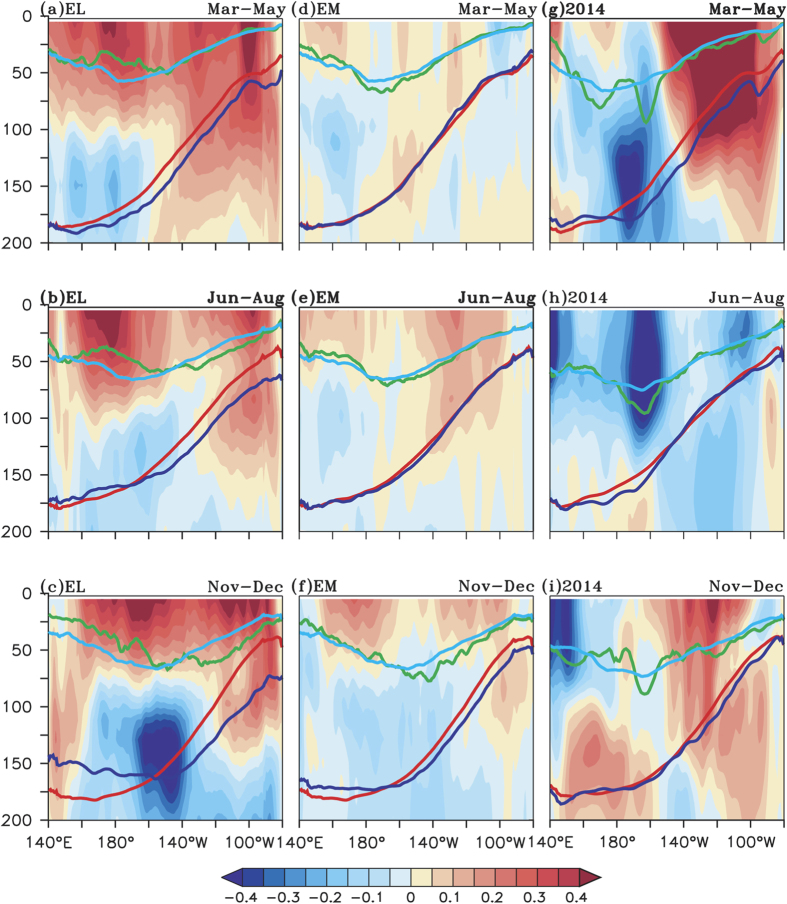
Depth longitude section showing zonal current anomalies (m s^−1^, shaded), the depth of the thermocline (m, red for climatological and blue for particular event) and mixed layer depth (m, light blue for climatology and green for the particular event during Mar–May of (**a**) Canonical El Niño (**d**) El Niño Modoki and (**g**) 2014. (**b,e,h**) same as (**a,d,g**) except for Jun–Aug. (**c,f,i**) same as (**a**,**d**,**g**) except for Nov–Dec. The depth of the 20 °C isotherm is considered as thermocline depth.

During an EL, anomalous westerly winds generate a downwelling Kelvin wave, accompanied by anomalous surface currents that transport warmer water to the east^23^,^48^. In other words, the processes of the anomalous advection of warmer water and a deepening of the thermocline anomalously warm the SST in the eastern Pacific Ocean. This in turn reinforces the weakening of the trade winds, and initiates positive feedbacks that result in a mature phase of canonical El Niño^47^,^49^,^50^. We show the zonal current anomalies for the upper 200 m in the equatorial Pacific between 2°S-2°N during MAM, JJA and Nov-Dec of EL, EM and TP (2014) in Fig. 4 (shaded). Strong anomalous eastward currents can be seen in the upper few hundred meters of water column during ELs as compared to the EMs and TP events (Fig. 4), with maximum intensity during boreal winter (Nov-Dec). Thus, throughout the EL, eastward transportation of warm water takes place, which deepens the thermocline in the east and shoals it in the west (Fig. 1a). But, in the case of EMs, relatively weak WWA induce weak anomalous eastward currents, as shown in Figs. 4d–f; the thermocline response is also weaker (Fig. 2b) as compared to that during an EL.

Importantly, during the 2014 TP event (Fig. 4g–i), eastward currents are seen in the eastern equatorial Pacific during Mar-May and during the mature phase Nov-Dec. The eastward currents are absent during boreal summer consistent with the absence of boreal summer WWA in the western Pacific. Stronger anomalous eastward zonal currents are seen during boreal winter of 2009 (Fig. S3c) in response to WWA (Fig. S1b) in the western-central Pacific.

To further verify the ocean dynamic response, we have analyzed the subsurface conditions, in the equatorial Pacific, mixed layer and thermocline variations during boreal spring (MAM), boreal summer (JJA) and boreal winter (ND) as shown in Fig. 4 & S3c, respectively. During the boreal spring of an EL, thermocline (blue line) is deeper in the east with slight shoaling in the west (Fig. 4a). The thermocline deepening in the east is enhanced during boreal summer and a sharp east-west gradient in thermocline anomalies is generated during the mature phase (Nov-Dec) of EL, with deepening of about 30 m in the east (blue-red) and shoaling of similar magnitude in the west. The sharp anomalous thermocline gradient enhances the Bjerknes feedback and amplifies the warm anomalies in the east. Since the thermocline is deeper in the east, it is decoupled from the surface layers and thus warm SSTA persist in the eastern tropical Pacific during EL.

In the case of the EM, the east-west thermocline slope (blue line) is close to the climatological position (red line) during boreal spring and summer and is slightly deepened in the east during the mature phase (Fig. 4d–f) generating a weak east-west thermocline slope anomaly. The mixed layer depth, as shown by green line in Fig. 4d–f, is anomalously closer to the thermocline (blue) during boreal spring and summer, and thus can easily interact with the thermocline. Thus the colder subsurface water is brought close to the surface as a result of upwelling by the anomalous easterly winds in the eastern equatorial Pacific (Fig. 2b) and results in cold SSTA in the eastern Pacific during boreal summer of EM (Fig. 4e). As a result, warm SSTA is maintained only in central equatorial Pacific and the surface wind converges to the warm central Pacific SST region, and ensures persistence of the warm SSTA in the central equatorial Pacific during EM event. It has to be noted that during boreal spring of TP event (Fig. 4g) the thermocline is deeper in the east similar to EL (Fig. 4a) indicating the preconditioning of the ocean by boreal spring of TP similar to EL. During boreal summer (Fig. 4h) the thermocline is close to climatological position similar to EM generating a basinwide warming pattern.

Thus, while the distinction between the EL and EM is seen in zonal current evolution, that between the EL and TP is manifested in the thermocline variations.

### Potential mechanism for distinct evolution of SST anomalies during TP events

To verify and ascertain the observational conjectures we made in the previous section relevant to anomalous evolution of the various ENSO types, in this section, we examine the results from our Ocean General Circulation Model experiments (see data and Methods for details of the experiments).

**Figure 5:**
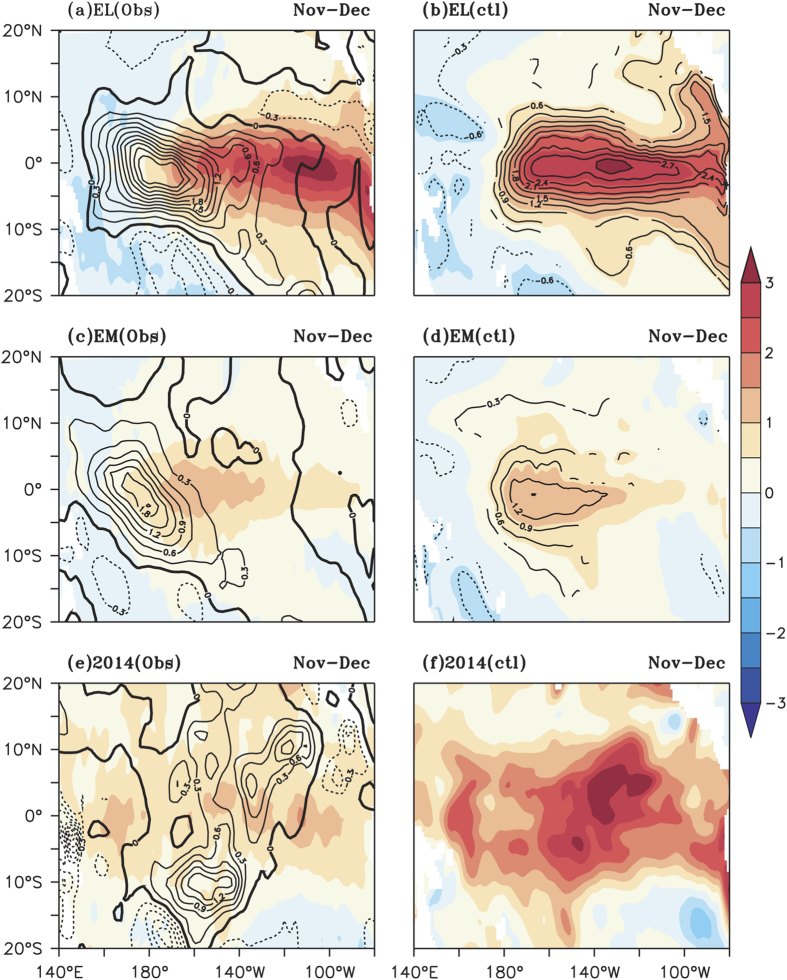
Composite SST anomalies (°C, shaded) for Nov-Dec of (**a**) canonical El Niño (**c**) El Niño Modoki and (**e**) 2014 from observation. The zonal wind stress anomalies (10^−2^ Nm^−2^) are shown as contours in Fig. (**a**,**c**,**e**). Composite SST anomalies (°C) from model simulation are shaded for Nov-Dec in (**b**,**d**,**f**). The anomalies above 90% confidence level from a two-tailed student’s t-test are shown as contours in (**b**,**d**).

The composited SSTA of EL, EM and TP during the Nov-Dec period obtained from the ctl experiment, are shown in Fig. 5, along with the corresponding observations. The anomalies above 90% confidence level (contour) from a two-tailed student’s t-test are shown for EL and EM from control experiment in Fig. 5b,d. The warm SSTA in the central and eastern tropical Pacific during Nov-Dec of EL are reasonably well represented in the ctl exp (Fig. 5b). The EM events with warm SSTA in the central tropical Pacific during Nov-Dec are also well represented in the ctl exp (Fig. 5d). During 2014 (Fig. 5e,f) and 2009 (Fig. S3a,b), a basinwide warming pattern is seen both in observations and model results. It can also be noted that the spatial pattern of wind stress anomaly (contour; 10^−2^ Nm^−2^, Fig. 5a,c,e) is also different between different El Niño events.

**Figure 6:**
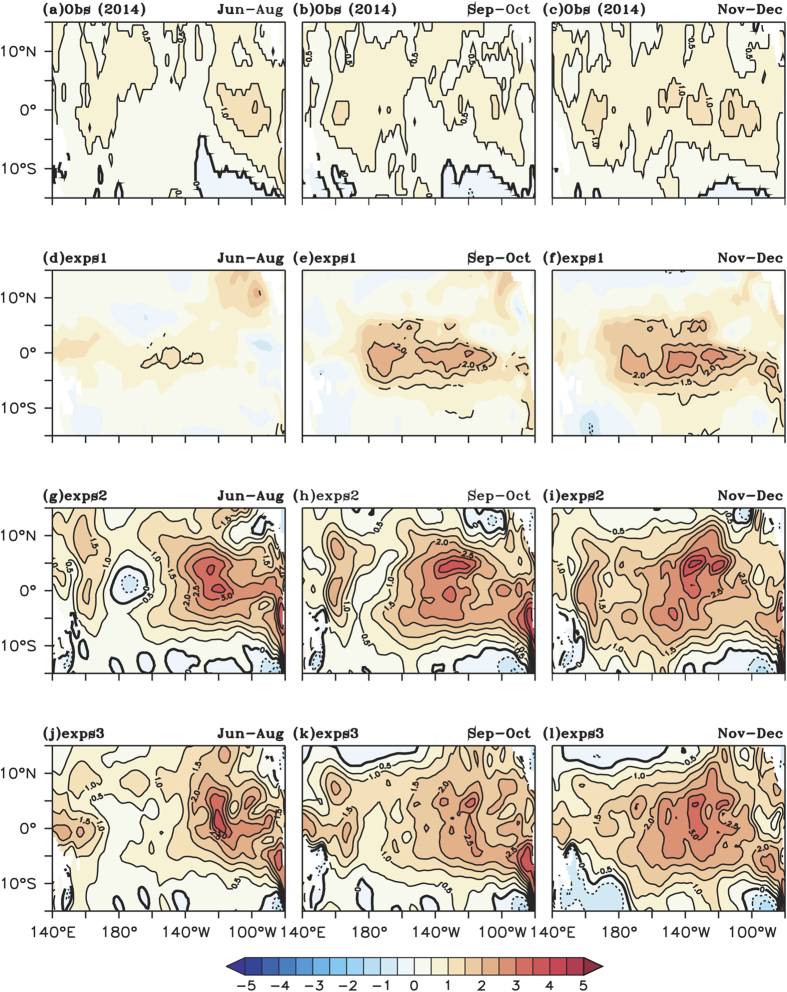
Sea surface temperature anomalies (°C) for Jun–Aug of (**a**) 2014 from observation (**d**) exps1 (**g**) exps2 and (**j**) exps3. (**b**,**e**,**h**,**k**) same as (**a**,**d**,**g**,**j**) except for Sep–Oct. Similarly, (**c**,**f**,**i**,**l**) same as (**a**,**d**,**g**,**j**) except for Nov–Dec. Significance values above 90% confidence level from two-tailed student’s t-test are shown as contours for exps1 in (**d**,**e**,**f**).

To understand the relevance and relative importance of ocean pre-condition and the atmospheric forcing on the El Niño evolution we have performed several sensitivity experiments (exps1-exps5). Briefly, in the exps1, exps2 and exps3, the ocean is initialized with January 1997 ocean condition. That is, the tropical pacific is preconditioned for a strong El Niño. For the three constituent ensembles of the exps1, the OGCM is forced with winds and shortwave and longwave fluxes from three strongest EM events (1994, 2002, and 2004) separately. In the exps2, the OGCM is forced with the 2014 and exps3 with 2009 wind and fluxes respectively. The spatial pattern of SST anomalies from these experiments along with observed anomalies during 2014 is shown in Fig. 6.

The ensemble mean of the simulated SSTA from the exps1, along with significant values above 90% confidence level from a student’s two tailed t-test (contour) are shown in Fig. 6d–f. The SST anomalies from exps2 and exps3 are shown in Fig. 6g–l respectively. It is interesting to note that a basinwide warming pattern, similar to that observed in Fig. 6a–c is seen in all three experiments (exps1, exps2 and exps3) commencing from boreal summer (Jun-Aug). These anomalies are different from control simulation of EL and EM. In the case of the EM, the control simulation shows significant warming in the central Pacific with cooling on both sides (Fig. 5d), while exps1 basinwide warming is seen (Fig. 6f). The anomalies in exps2 and exps3 are slightly higher than exps1; one of the possible reasons for the higher magnitude may be the changes in the forcing. The Corrected Interannual Forcing (CIAF, Large & Yeager^51^) is available till 2009, and exps2 and exps3 is forced with NCEP winds and fluxes.

The anomalies of zonal wind (shaded) and wind stress (contour) are shown in Fig. S4. It has to be noted that spatial pattern of zonal wind anomalies are different between these experiments. Anomalous westerlies are seen in the central eastern equatorial Pacific during 2009 and central Pacific during 2014 and are not seen beyond dateline during El Niño Modoki events. However, the sensitivity experiments exps1, exps2 & exps3 forced with same ocean initial condition (1997) and winds for EM events, 2014 and 2009 show a similar pattern of SST anomalies with a basinwide warming (TP). This indicates that strong ocean pre-condition and weak winds will result in a TP event. It is also seen that easterly wind anomalies prevail near the date line on the equator during boreal summer of both the TP event; they can also cause westward extension of SST warming. Another point to be noted is that the sensitivity experiments are performed with an ocean GCM and the boundary forcing dominate as compared to initial condition, which is one of the limitations with Ocean GCM sensitivity experiment.

Another set of experiment (exps4) has also been performed with three ensembles, each with the oceanic and atmospheric initial conditions of three strongest EM events (1994, 2002 & 2004) separately. The ensemble mean from these three major EM events is shown for June-August (JJA), Sept-Oct (SO) and Nov-Dec (ND) in Fig. S5a-c and significance values above 90% confidence level from a two-tailed student’s t-test are shown as contour. During JJA and SO, warm SSTA are seen in the central equatorial Pacific, flanked by colder SSTA on both sides similar to EM. The warming is enhanced and extends to eastern Pacific similar to EM during the mature phase. Thus the exps4 with weak ocean condition and weak atmospheric forcing resulted in a SSTA pattern similar to EM.

We have also performed exps5 with three ensembles. In each of these, the model is forced with 1997 winds i.e. a strong atmospheric forcing; But each of these, has weak oceanic pre-condition associated with the three EM (1994, 2002 and 2004) events. The ensemble means of SSTA for JJA, SO and ND are shown in Fig. S5d-f. The SSTA show large positive anomalies with maximum anomalies of more than 5 °C in the central Pacific and cooling in the western Pacific. These anomalies are different from a TP case because for a TP event basinwide warming is seen and it is extending from western to eastern Pacific. Also, the SSTA from exps5 is different from the control simulation EL (Fig. 5b), which shows significant warming in central and eastern Pacific and cooling in the western Pacific. The exps5, on the other hand, shows a strong anomalous warming in the central Pacific (Fig. S5f). Thus the exps5 resulted in a pattern which is not representative of any of the three events EM, EL or TP.

The results from the sensitivity experiments reveal that strong ocean pre-condition and weak WWA will generate a TP and weak ocean condition and weak WWA will result in EM. Thus the intensity of ocean pre-condition and that of the westerly wind anomalies are crucial for the distinct evolution of El Niño events.

## Discussion

Different El Niño types has been observed in the recent decades with warm anomalies in the eastern Pacific, known as canonical El Niño (EL) and warming in the central equatorial Pacific, El Niño Modoki (EM). Among these, a basinwide warming pattern (TP) has been only seen during 2009 and during 2014. Nonetheless, owing to the distinct basinwide warming patterns and teleconnections^15^, the 2009 and 2014 events are classed as separate from the two known types. The location of the warm SSTA during an El Niño type is very crucial in determining the global climate impacts. The El Niño Modoki events significantly influence the temperature and precipitation over many parts of the globe in ways quite different from the conventional El Niño events^7^,^9^. The basinwide warming pattern of 2009 had caused severe drought over India, and heat waves in Europe, etc. Similarly during 2014 also Indian summer monsoon rainfall was anomalously deficit. Thus, it is very important to understand the nature of the Pacific warm event types, and the relevant generating mechanisms.

In experiments carried out in this study, the ocean IC may not exactly match with the surface forcing, which is a quintessential limitation with such sensitivity experiments with any OGCM. Keeping this in mind, we plan to analyse long term climate simulations with coupled GCMS, such as the CMIP5 outputs for further understanding. In addition, we also plan to carry out further sensitivity experiments with the OGCM to explore the sensitivity of the simulated flavours to the localization of the WWE to a particular sub-region of the tropical pacific.

Keeping this in mind, we have performed a detailed analysis of observed datasets and reanalysis products to understand the possible cause. Numerical simulation experiments have also been performed to substantiate our findings. The results reveal that the locations of warm SST anomalies are modulated by the intensity and seasonality of westerly wind anomalies (WWA) and ocean pre-conditioning. Relatively stronger ocean pre-conditioning and stronger boreal spring (Mar-May) to summer (June-September) westerly wind anomalies (WWA) can generate anomalously strong east-west gradient in the equatorial thermocline anomaly which can amplifies the SST anomalies through Bjerknes feedback during EL. Relatively weaker WWA and preconditioning during EM, on the other hand can lead to weak east-west thermocline slope. This facilitates the subsurface water to interact with the surface water and damp the warming in the east. Thus, warming is primarily confined to the central equatorial Pacific, which amplifies through surface wind convergence. During the two TP events of 2009 and 2014, though the tropical Pacific Ocean was pre-conditioned similar to an EL, the lack of WWA in the western-central Pacific has likely resulted in a basinwide warming pattern.

The results from our study provide an important insight about the possible cause for distinct warming pattern during different El Niño types, and a clue in understanding the type of the evolving El Niño at least two seasons ahead. Given the challenges for climate models to predict the distinction between different El Niño types^52^,^53^, accurate prediction of the strength of zonal winds and ocean condition during boreal spring will, therefore, help in predicting the flavour of the predicted El Niño signal more accurately, and prepare for its potential impacts better.

## Methods

### Data

The datasets used include the Met Office Hadley Centre Global Sea Ice and Sea Surface Temperature (HadISST; Rayner *et al.*^54^), surface winds from National Centre for Environmental Prediction (NCEP, Kalnay *et al.*^55^), for the period 1950–2014, and the subsurface datasets from Simple Ocean Data Analysis (SODA, Carton *et al.*^56^,) for the period 1958–2008, and Global Ocean Data Assimilation (GODAS, Behringer and Xue^57^). Following general convention, we interpret the depth of 20 °C isotherm (D20) as the thermocline depth. Further, the mixed layer depth is estimated as the depth at which the subsurface cooler than the surface by 0.2 °C. The warm water volume from TAO data for the period 1980–2014 is taken from NOAA /PMEL (http://www.pmel.noaa.gov/tao/elnino/wwv/).

We distinguish the EL (EM) events based on the criteria that the amplitude of the NIÑO3 (ENSO Modoki index) index exceeds one seasonal standard deviation for boreal summer through following boreal winter^34^. While Niño3 is the area-averaged sea surface temperature anomaly over the region (150°W-90°W, 5°N-5°S); and El Niño Modoki index is defined as EMI = [SSTA]_A_−0.5 * [SSTA]_B_−0.5 * [SSTA]_C_ where the square bracket represents the area averaged SSTA over each of the region A (165°E-140°W, 10°S-10°N), B (110°W-70°W, 15°S-15°N), and C (125°E-145°E, 10°S-20°N).

Accordingly, 1957, 1965, 1972, 1982 and 1997 are designated as the EL events and 1967, 1977, 1991, 1994, 2002 and 2004 as the EM events. Owing to the distinct basinwide warming patterns, the 2009 and 2014 events are classed as separate from the two known flavours. All the anomalies are computed for by subtracting the mean for the period 1950–2014 expect for SOAD and GODAS. For SODA (GODAS) climatology is computed for the period 1958–2008 (1980–2014).

### Ocean Model

We also carry out control and sensitivity experiments with the version 4p1 of the Modular Ocean Model (MOM4p1); see Griffies *et al.*^58^, for details. The model had been spun-up by initializing the model with the annual climatologies of temperature and salinity from Levitus *et al.*^59^, and forced with climatological forcing derived from CORE (Common Ocean-ice Reference Experiments), and then integrated for another 120 years to reach a steady state. From this steady state, an interannual integration was carried out for the period 1948–2014 using Corrected Interannual Forcing (CIAF, following that of Large & Yeager^51^) and NCEP reanalysis data. We refer to this interannual experiment as the control experiment – designated as ctl exp. We use the results from this experiment to estimate the model climatology and also to understand the ocean subsurface dynamics during different El Niño types. In addition, sensitivity experiments (exps1-exps5) have been performed using January ocean initial condition (IC) for particular experiment and the model is integrated for a 12 month period. The details about the sensitivity experiments are described in Table 1. The atmospheric forcing in all sensitivity experiments are climatological forcing except for surface winds and long wave and shortwave radiation. All the other fluxes are computed by the model. Importantly, the ocean initial condition in exps1, exps2 and exps3 pertains to the January 1997 conditions. *That is the ocean initial condition in these experiments is therefore, conducive for the development of a very strong El Niño* such as that seen in the year1997. However, in both the exps1 and exps4, the OGCM is forced with winds and fluxes for three strong El Niño Modoki events (1994, 2002 and 2004) separately and the ensemble mean in presented in Fig. 6 and Fig. S5. The ocean IC in exps4 is also from 3 ensembles of EM (1994, 2002 and 2004). The exps5 is initialized with ocean IC for 1994, 2002 and 2004 separately and is forced with 1997 winds and fluxes.

## Additional Information

**How to cite this article**: Jadhav, J. *et al.* On the possible cause of distinct El Niño types in the recent decades. *Sci. Rep.* **5**, 17009; doi: 10.1038/srep17009 (2015).

## Supplementary Material

Supplementary Information

## Figures and Tables

**Table 1:** Details of Model sensitivity experiment.

Exp	IC (Initial condition)	BC (Boundary condition)	Ocean Pre-condition/Atmospheric Forcing	Result
Exps1 (3 ensembles)	1997	El Niño Modoki winds and fluxes for 1994,2002 and 2004	Strong ocean pre-condition and weak atmospheric forcing	TP event
Exps2	1997	2014 wind and fluxes	Strong ocean pre-condition and weak atmospheric forcing	TP event
Exps3	1997	2009 wind and fluxes	Strong ocean pre-condition and weak atmospheric forcing	TP event
Exps4 (3 ensembles)	1994, 2002 and 2004 El Niño Modoki ocean IC	El Niño Modoki winds and fluxes for 1994, 2002 and 2004	Weak ocean pre-condition and weak atmospheric forcing	EM event
Exps5 (3 ensembles)	1994, 2002 and 2004 El Niño Modoki ocean IC	1997	Weak ocean pre-condition and strong atmospheric forcing	Unrealistic case (spatial pattern of SST anomalies different from EL, EM and TP events)
