# Supplementary material for: On the possible cause of distinct El Niño types in the recent decades
Source: Sci Rep. 2015 Nov 24;5:17009. doi: 10.1038/srep17009 (PMC4657059; doi:10.1038/srep17009)
Supplement: Supplementary Information [file srep17009-s1.pdf]

On the possible cause of distinct El Niño types in the recent decades

Jyoti[1] J, Swapna[1*] P, Shamal Marathe[1] and K. Ashok[2, 3]

[1]Centre for Climate Change Research, Indian Institute of Tropical Meteorology, India

[2] University of Hyderabad, India

[3] On lien from IITM, Pune, India

Pune 411008, India.

***Corresponding Author Address**

Dr. Swapna Panickal

Centre for Climate Change Research,

Indian Institute of Tropical Meteorology,

Pune 411008, INDIA.

Tel: +9125904538

Email: swapna@tropmet.res.in

Supplementary Figures

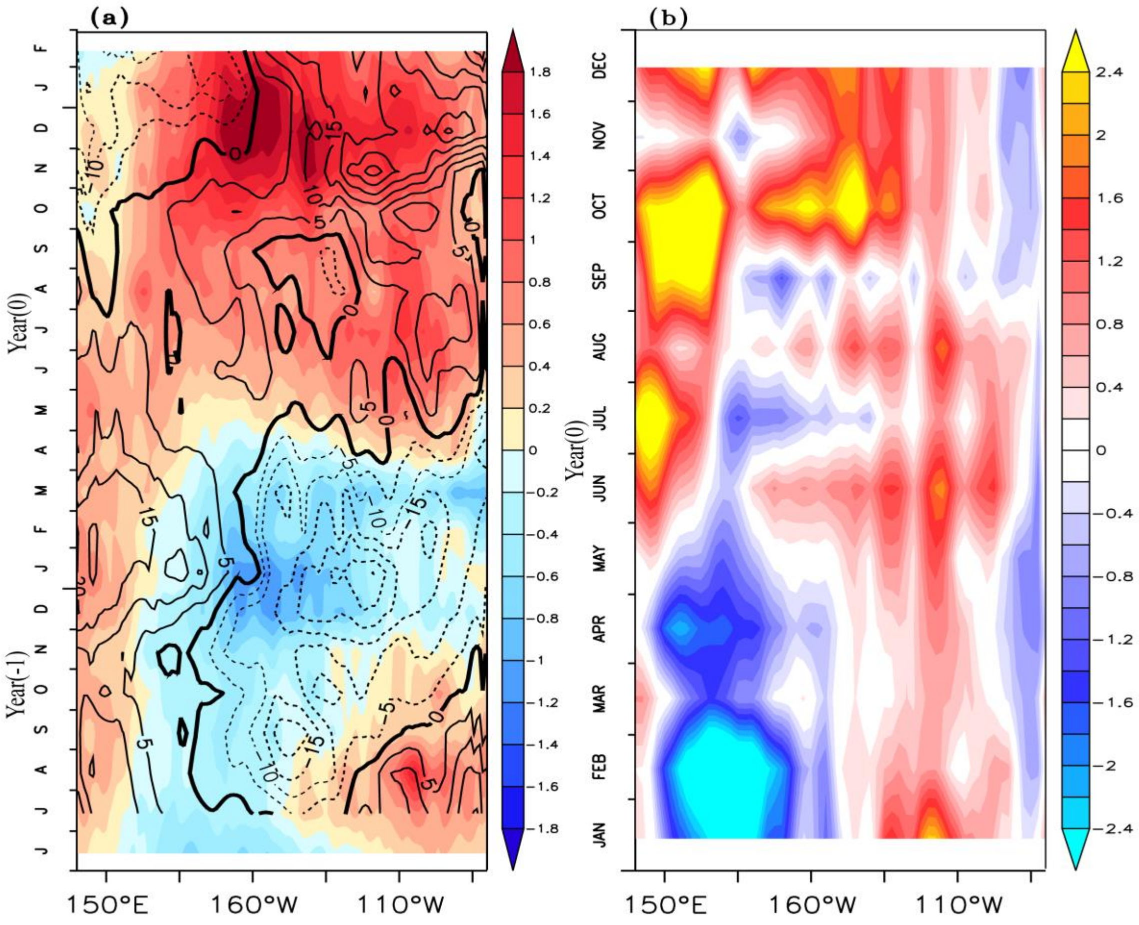

Figure S1

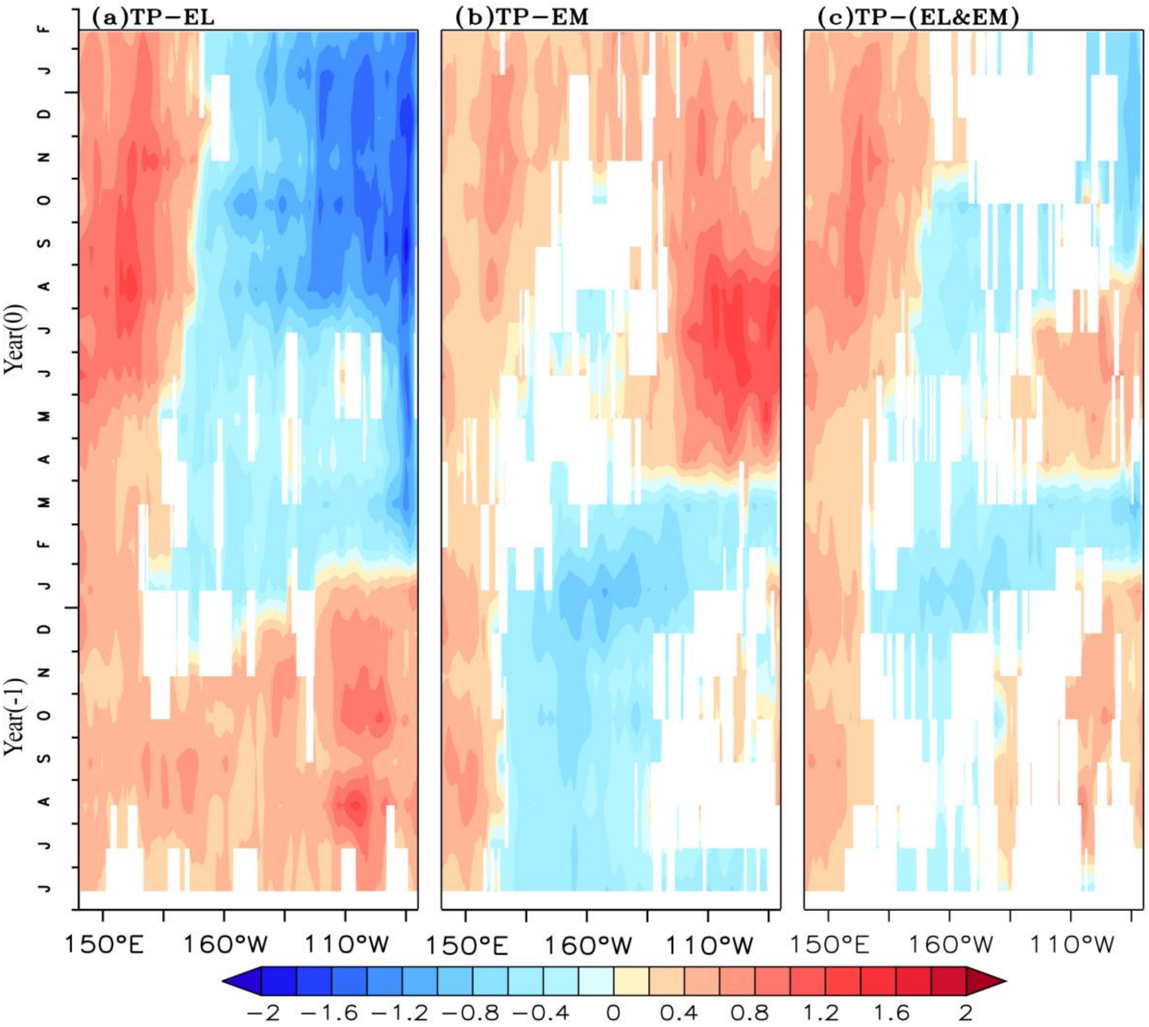
(a)TP−EL
(b)TP−EM
(c)TP−(EL&EM)
Year(0)
Year(-1)
J J A S O N D J F M A M J J A S O N D J F
150°E
160°W
110°W
−2
−1.6
−1.2
−0.8
−0.4
0
0.4
0.8
1.2
1.6
2

Figure S2

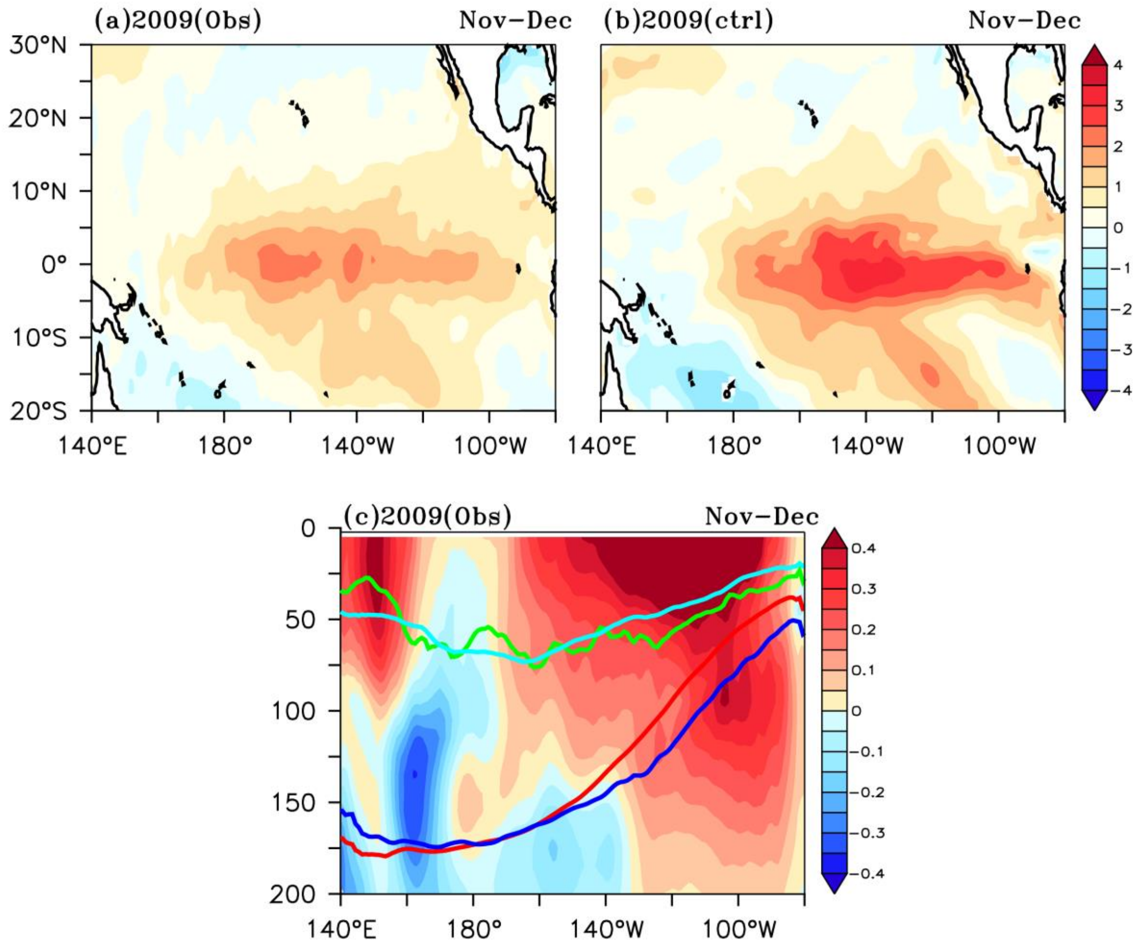
(a)2009(Obs)
Nov-Dec
(b)2009(ctrl)
Nov-Dec
30°N
20°N
10°N
0°
10°S
20°S
140°E
180°
140°W
100°W
4
3
2
1
0
-1
-2
-3
-4
(c)2009(Obs)
Nov-Dec
0
50
100
150
200
0.4
0.3
0.2
0.1
0
-0.1
-0.2
-0.3
-0.4

Figure S3

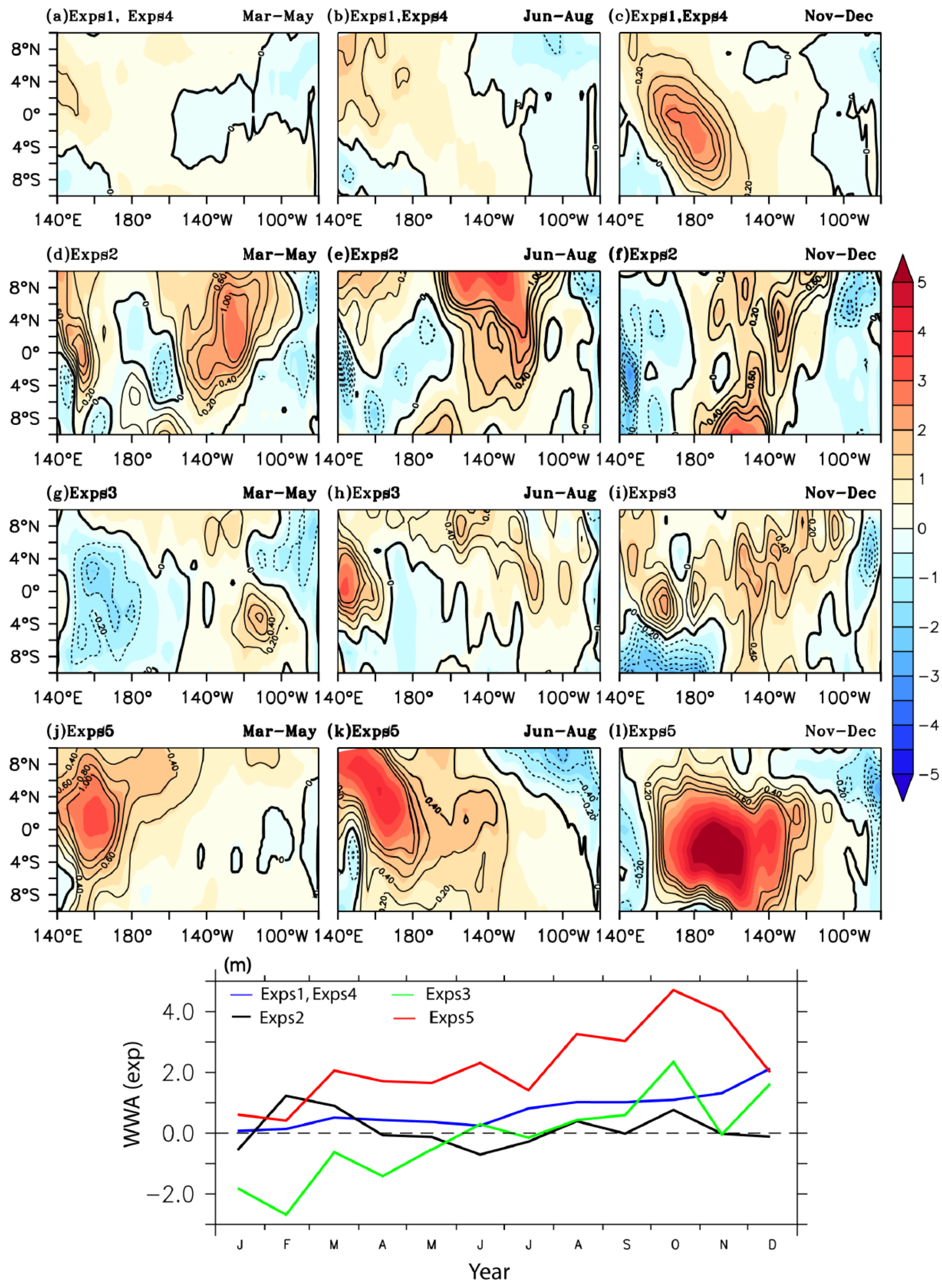
(a)Exps1, Exps4
Mar–May
(b)Exps1,Exps4
Jun–Aug
(c)Exps1,Exps4
Nov–Dec
(d)Exps2
Mar–May
(e)Exps2
Jun–Aug
(f)Exps2
Nov–Dec
(g)Exps3
Mar–May
(h)Exps3
Jun–Aug
(i)Exps3
Nov–Dec
(j)Exps5
Mar–May
(k)Exps5
Jun–Aug
(l)Exps5
Nov–Dec
8°N
4°N
0°
4°S
8°S
140°E
180°
140°W
100°W
5
4
3
2
1
0
−1
−2
−3
−4
−5
(m)
Exps1, Exps4
Exps2
Exps3
Exps5
WWA (exp)
4.0
2.0
0.0
−2.0
J
F
M
A
M
J
J
A
S
O
N
D
Year

Figure S4

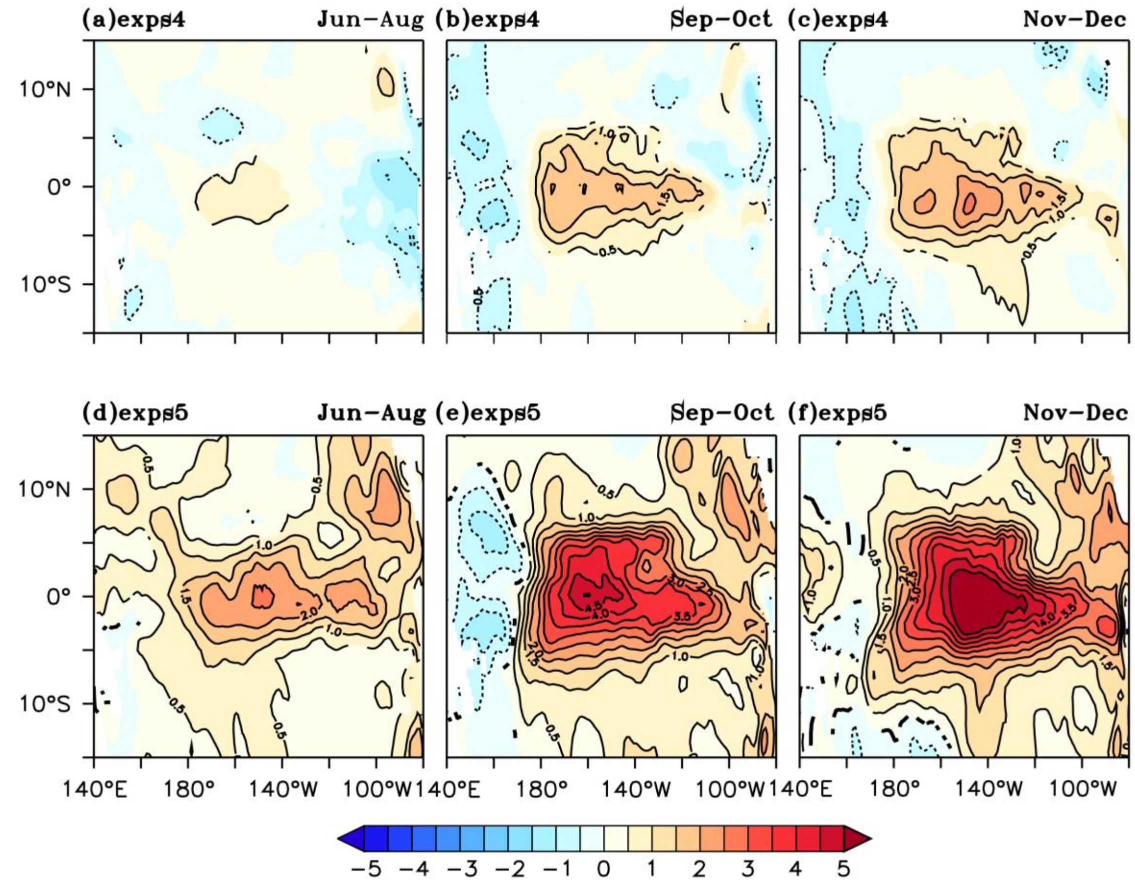

(a)exps4
Jun−Aug
(b)exps4
Sep−Oct
(c)exps4
Nov−Dec
(d)exps5
Jun−Aug
(e)exps5
Sep−Oct
(f)exps5
Nov−Dec
10°N
0°
10°S
140°E
180°
140°W
100°W
−5
−4
−3
−2
−1
0
1
2
3
4
5

Figure S5

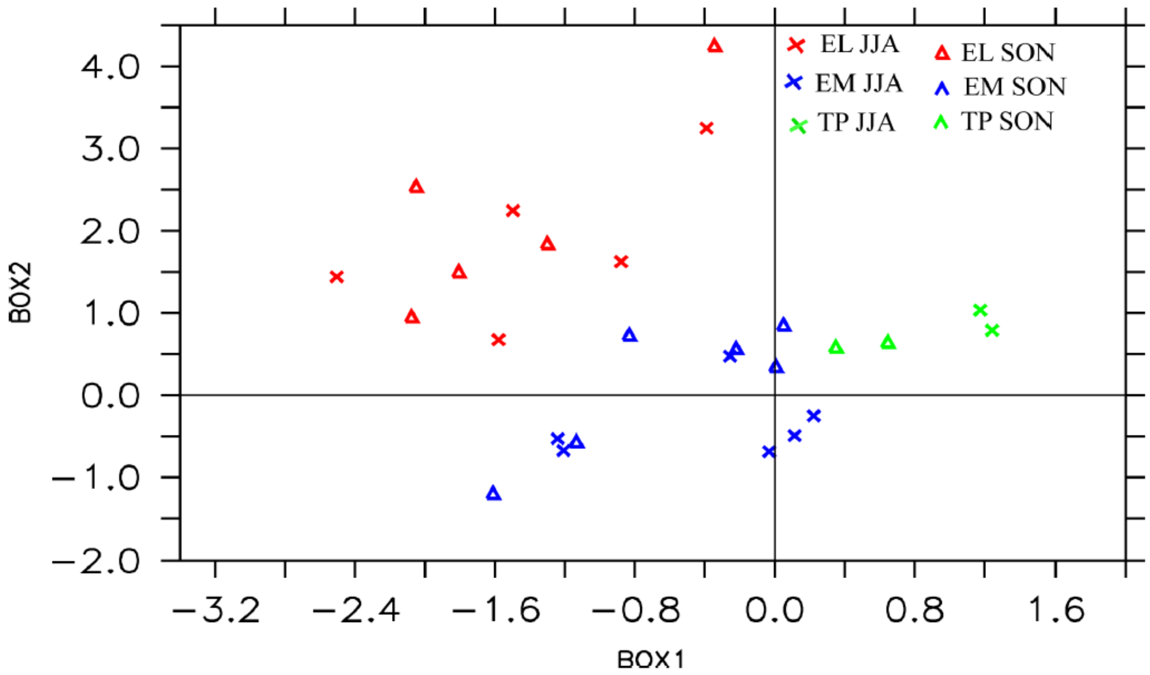

Figure S6

Figure Legends

Figure S1. (a) Time-longitude plot showing composite anomalies of sea surface temperature (^{0}C, shaded) and 20^{0}C isotherm depth (m, contour) averaged between 5^{0}S-5^{0}N for 2009. (b) same as (a) except for zonal wind anomalies (m s^{-1}).

Figure S2. Time-longitude plot showing the difference in composite anomalies of sea surface temperature (^{0}C, shaded) averaged between 5^{0}S-5^{0}N for (a) TP and canonical El Niño events (b) TP and El Niño Modoki events (c) TP and canonical El Niño as well as El Niño Modoki events. The TP events of 2014 and 2009 are considered for calculating the difference map.

Figure S3. SST anomalies (^{0}C, shaded) composited for Nov-Dec of (a) 2009 from observation. (b) same as (a) showing SST anomalies (^{0} C) except from model simulation. (c) The depth-longitude section showing anomalous zonal current (ms^{-1}; shaded), the depth of the thermocline (m, red for climatological and blue for particular event) and mixed layer depth

(m, light blue for climatology and green for the particular event) for 2009 from observation during Nov-Dec.

Figure S4.Zonal wind anomalies (shaded; ms^{-1}) and zonal wind stress anomalies (contour; 10^2 Nm^{-2}) for Mar-May of (a) exps1, exps4 (d) exps2 (g) exps3 and (j) exps5. (b), (e), (h), (k) same as (a), (d), (g), (j) except for Jun-Aug. Similarly, (c), (f), (i), (l) same as (a), (d), (g), (j) except for Nov-Dec. (m) Time series showing the seasonal evolution of zonal wind anomalies ($m\ s^{-1}$) over equatorial western Pacific (150^0E-160^0W, 5^0S-5^0N) region used for the forcing of exps1&exps4(blue), exps2(black), exps3(green), exps5(red).

Figure S5. Sea surface temperature anomalies (^{0}C) for Jun-Aug of (a) exps4. (d) exps5. (b), (e) same as (a), (d) except for Sep-Oct. Similarly, (c), (f) same as (a), (d) except for Nov-Dec. Significance values above 90% confidence level from a two tailed student's t-test are shown as contour for exps4 and exps5.

Figure S6. Scatter plot of standardised SST anomalies averaged in western and eastern Pacific boxes (Box1: 90^0W-80^0W; 5^0S-5^0N and Box2:125^0E-145^0E; 5^0S-5^0N) for EL (red), EM (blue) and TP (green) events during two consecutive seasons of JJA and SON.
